# Injection Site Matters: A Comparative Analysis of Transpulmonary Thermodilution via Simultaneous Femoral and Jugular Indicator Injections under Veno-Venous Extracorporeal Membrane Oxygenation Therapy

**DOI:** 10.3390/jcm13082334

**Published:** 2024-04-17

**Authors:** Sabrina Kopp, Johannes Windschmitt, Lena Schnauder, Thomas Münzel, Karsten Keller, Susanne Karbach, Lukas Hobohm, Philipp Lurz, Ingo Sagoschen, Johannes Wild

**Affiliations:** 1 Department of Cardiology, Cardiology I, University Medical Center Mainz, 55131 Mainz, Germany; 2 German Center for Cardiovascular Research (DZHK), Partner Site RheinMain, 55131 Mainz, Germany; 3 Center for Thrombosis and Hemostasis (CTH), University Medical Center Mainz, 55131 Mainz, Germany; 4 Department of Internal Medicine and Nephrology, University Hospital Marburg, 35043 Marburg, Germany

**Keywords:** hemodynamic monitoring, extracorporeal membrane oxygenation, transpulmonary thermodilution, PiCCO

## Abstract

**Background:** The use of veno-venous extracorporeal membrane oxygenation (vv-ECMO) in acute lung failure has witnessed a notable increase. The PiCCO system is frequently used for advanced hemodynamic monitoring in this cohort. Our study aimed to investigate whether the choice of indicator injection site (jugular vs. femoral) in patients undergoing vv-ECMO therapy affects transpulmonary thermodilution (TPTD) measurements using the PiCCO^®^ device (Pulsion Medical Systems SE, Munich, Germany). **Methods**: In a retrospective single-center analysis, we compared thermodilution-derived hemodynamic parameters after simultaneous jugular and femoral injections in 28 measurements obtained in two patients with respiratory failure who were undergoing vv-ECMO therapy. **Results**: Elevated values of the extravascular lung water index (EVLWI), intrathoracic blood volume index (ITBVI) and global end-diastolic volume index (GEDVI) were observed following femoral indicator injection compared to jugular indicator injection (EVLWI: 29.3 ± 10.9 mL/kg vs. 18.3 ± 6.71 mL/kg, *p* = 0.0003; ITBVI: 2163 ± 631 mL/m^2^ vs. 806 ± 125 mL/m^2^, *p* < 0.0001; GEDVI: 1731 ± 505 mL/m^2^ vs. 687 ± 141 mL/m^2^, *p* < 0.0001). The discrepancy between femoral and jugular measurements exhibited a linear correlation with extracorporeal blood flow (ECBF). **Conclusions**: In a PiCCO®-derived hemodynamic assessment of patients on vv-ECMO, the femoral indicator injection, as opposed to the jugular injection, resulted in an overestimation of all index parameters. This discrepancy can be attributed to mean transit time (MTt) and downslope time-dependent (DSt) variations in GEDVI and cardiac function index and is correlated with ECBF.

## 1. Introduction

Extracorporeal membrane oxygenation therapy (ECMO) has become an integral part of advanced intensive care therapy in recent years. Veno-venous cannulation (vv-ECMO) serves to establish an extracorporeal circuit for the therapy of acute lung failure. This can be regarded as the ultima ratio or as a planned alternative therapy for acute lung failure. The use of vv-ECMO for lung replacement increased in Germany by 236%, from 825 cases in 2007 to 2768 cases in 2018, as the data from the Federal Statistical Office have shown [1]. Due to the COVID-19 pandemic and the resulting massive increase in COVID-19-related lung failure, the use of this procedure has experienced new highs. Thus, 3397 COVID-19 patients were treated with vv-ECMO in Germany from March 2020 to May 2021 [2]. The therapy is no longer reserved for an exclusive group of tertiary care hospitals but was performed in 231 intensive care units in Germany during this study period [1].

Due to the severe impact on a patient’s hemodynamics, the optimization of extracorporeal blood flow adapted to cardiac output is important for effective extracorporeal oxygenation requiring adequate hemodynamic monitoring [3]. Furthermore, fluid management in acute respiratory failure is crucial to minimize organ damage. For optimized fluid management and determination of cardiac output, hemodynamic monitoring, including pulse contour analysis and indicator dilution techniques, is recommended for critically ill patients [4]. Transpulmonary thermodilution (TPTD) can be used to determine cardiac output (CO), global end-diastolic volume index (GEDVI), intrathoracic blood volume index (ITBVI), and extravascular lung water index (EVLWI).

Studies have already shown that TPTD-derived parameters with femoral indicator injections have led to a significant overestimation of the GEDVI compared to jugular indicator injections [5]. Concerning the injection site and investigating TPTD in patients undergoing vv-ECMO therapy, Herner et al. not only illustrated how all TPTD-derived parameters changed after the initiation of vv-ECMO therapy, but they also noted that the parameters measured in patients after femoral injections were significantly higher than those after jugular injections. Multivariate analyses revealed a significant independent correlation between increased GEDVI, EVLWI, and the femoral injection site [6]. Due to this and further evidence suggesting relevant differences caused by the site of indicator injection [5], we performed measurements of both sites (jugular/femoral) in patients with jugular and femoral access in routine clinical practice and interpreted the results in a clinical context.

The aim of our pilot study was to investigate whether the site of injection—femoral vs. jugular—has a significant influence on the comparability and validity of TPTD-derived measurements. Although there are several publications on the topic of managing advanced hemodynamic monitoring in patients with extracorporeal support systems [5,7,8], this is the first analysis of hemodynamic patterns using the PiCCO^®^ system at different measurement sites simultaneously in the same patient with ongoing vv-ECMO therapy.

## 2. Materials and Methods

### 2.1. Patient Population

In our retrospective analysis (Ethics approval Nr. 2023-17036, Ethics committee Rhineland-Palatinate), we included patients of the medical intensive care unit at the Centre for Cardiology at the University Medical Centre Mainz treated with vv-ECMO who had both jugular and femoral central access and a Pulse Contour Cardiac Output system (PiCCO^®^, Pulsion Medical Systems SE, Munich, Germany). To detect suitable data sets, we evaluated all patients treated with respiratory failure and vv-ECMO therapy in our intensive care unit (ICU) from 1 January to 31 December 2022. We included 28 simultaneous TPTD measurements from two different patients.

The standard cannulation for V_f_-V_j_-ECMO was a femoral withdrawal cannula (23 French, 38 cm) and a jugular return cannula (19 French, 15 cm). The jugular access for indicator injection was a CVC (Arrow DE-14955-CV), and the femoral indicator injection access was a CDC (Achim Schulz-Lauterbach GmbH TLK12/20, Iserlohn, Germany). For the following thermodilution, an arterial PiCCO^®^ catheter (Getinge PV2015L20-A) was placed through the femoral artery in the descending aorta.

Further inclusion criteria were that all patients were sedated, on controlled ventilation, and not connected to any other extracorporeal device or continuous renal replacement therapy (CRRT). The retrospective observation period was set to twelve months. The indication for vv-ECMO therapy, as well as the subsequent treatment, was carried out according to current guidelines [9,10].

### 2.2. Principle of TPTD Measurements with the PiCCO Device

To perform TPTD measurements, a bolus of 20 mL of cold, isotonic saline is injected through a CVC or CDC. A sensor at the injection site registers the drop in temperature and starts the measurement process. The cold bolus moves through the right atrium, right ventricle, the lungs, the left atrium, and left ventricle; enters the systemic circulation; and is detected by an arterially inserted catheter. The consecutive change in blood temperature is detected by a thermistor-tipped catheter, which is usually placed through the femoral artery in the descending aorta. In this way, a thermodilution curve is recorded, and with its help, the cardiac output can be determined. Using the thermodilution curve, the patient’s volume parameters can be calculated semi-automatically with the help of various algorithms [11]. Cardiac parameters (cardiac output (CO), cardiac index (CI), cardiac functional index (CFI)), as well as parameters of blood volume and edema like GEDV, ITBV, and EVLW, are calculated using three main values derived from a contour analysis of the thermodilution curve: mean transit time (MTt), downslope time (DSt), and area under the curve (AUC). MTt describes the time until half of the injected saline bolus has passed. The DSt describes the duration of the exponential decrease in the thermodilution curve. The AUC reflects the estimated CO. The difference between MTt and DSt multiplied by CO results in the GEDV. The EVLW can be calculated from the difference of the ITTV (MTt × CO) and ITBV (1.25 × GEDV). In the PiCCO^®^ algorithm, real bodyweight is used to calculate the cardiac parameters. For volume parameters, on the other hand, the predicted bodyweight (PBW) and the predicted body surface area (BSA) are used.

### 2.3. Calculations

Using PiCCO^®^-derived parameters, EVLWI and ITBVI, we calculated EVLW and ITBV by multiplication with PBW and BSA. BSA was calculated with the Du Bois formula based on the real body weight for cardiac parameters and based on the PBW for all other parameters. The sum of these two parameters results in the intrathoracic thermal volume (ITTV). We calculated GEDV by dividing ITBV by 1.25. The pulmonary thermal volume (PTV) was calculated from the difference between ITTV and GEDV. Finally, MTt was calculated as a quotient of ITTV and CO, and DSt was calculated as a quotient of PTV and CO. For further statistical analyses, extracorporeal blood flow (ECBF) was indexed to BSA. Due to the extended times (MTt and DSt), not every measurement provided results for every parameter, as only parameters that pass an internal reliability algorithm are provided by the PiCCO^®^ software. This led to a reduction in the number of parameters provided. We show all the data provided to us by the PiCCO^®^ software as individual values in each figure.

### 2.4. Statistical Analysis

Descriptive statistics are given as mean values and standard deviations or as percentages of 100%. Normality was assessed by using a Kolmogorov–Smirnov normality test. To compare dependent measurements, we used a paired *t*-test or Wilcoxon signed-rank test, as appropriate. We used Pearson correlation followed by linear regression analysis. Statistical analyses were performed using Prism Version 9.5.0 (525), 8 November 2022 (GraphPad Software, San Diego, CA, USA). The level of significance was set at *p* < 0.05.

## 3. Results

A total of 28 extended hemodynamic measurements from two patients, using the TPTD method, were included in the analysis. The demographic and clinical characteristics of the patients are presented in Table 1. There were no relevant comorbidities.

**Figure 1:**
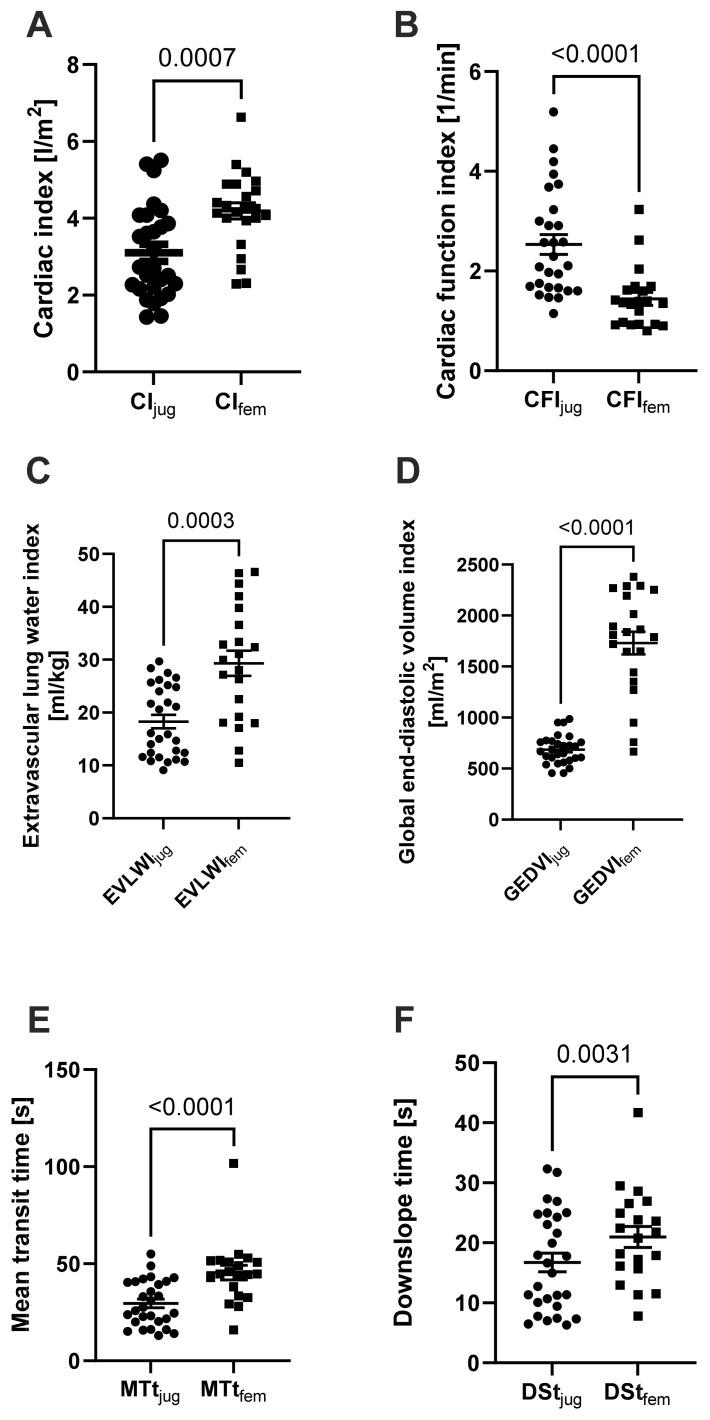
Comparison of TPTD-derived parameters from jugular or femoral indicator injections. (**A**) Cardiac index (CI, n = 23), (**B**) cardiac function index (CFI, n = 21), (**C**) extravascular lung water index (EVLWI, n = 21), (**D**) global end-diastolic volume index (GEDVI, n = 21), (**E**) mean transit time (MTt, n = 20), and (**F**) downslope time (DSt, n = 20) and paired *t*-tests. Data are reported as single values with median and SEM; *p*-values are shown above the brackets.

Cardiac function parameters, CO and CI, provided significantly higher measured values after the femoral indicator injection, whereas the CFI was significantly lower in measurements after the femoral indicator injection than after the jugular injection (Figure 1 and Supplementary Table S1). There were also significant differences in volume parameters; the femoral indicator injection yielded significantly higher results for EVLWI, ITBVI, and GEDVI. For EVLWI, we found a factor of 1.6 (*p* = 0.0003); for ITBVI, we found a factor of 2.7 (*p* < 0.0001); and for GEDVI, we found a factor of 2.5 (*p* < 0.0001) compared to results after jugular injections. Calculated times for MTt and DSt showed longer times in the measurements after the femoral indicator injection. MTt was 1.5 times (*p* < 0.0001) as long and the DSt was 1.3 times (*p* = 0.0031) as long as in the corresponding measurements with the jugular indicator injection.

**Figure 2:**
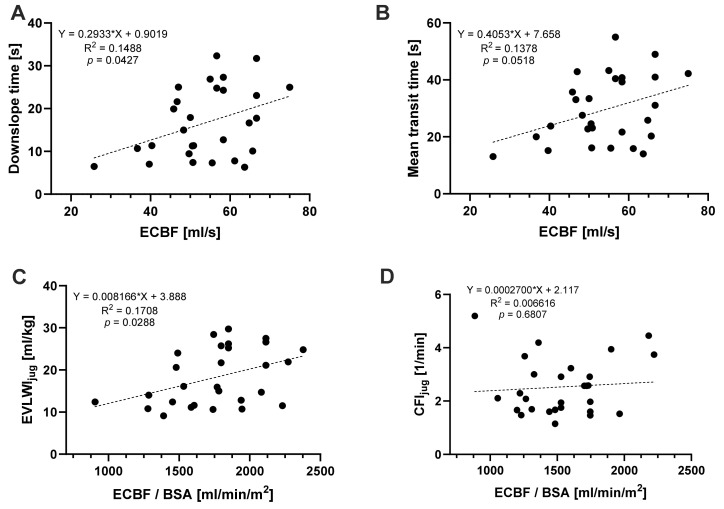
Simple linear regression model (n = 28) of the PiCCO®-derived jugular measurements as a function of the extracorporeal blood flow indexed to body surface area. (**A**) Downslope time, (**B**) mean transit time, (**C**) extravascular lung water index derived by jugular injection as a function of the extracorporeal blood flow indexed to body surface area, and (**D**) cardiac function index as a function of the extracorporeal blood flow indexed to body surface area.

In addition, we analyzed the correlation between TPTD-derived measurements after a jugular indicator injection and ECBF at the time of the measurement (Figure 2). We found a significant correlation between ECBF and DSt (*p* = 0.0427, R^2^ = 0.1488), as well as EVLWI (*p* = 0.0288, R^2^ = 0.1708). MTt and the cardiac function parameter CFI showed no significant correlation with ECBF.

**Figure 3:**
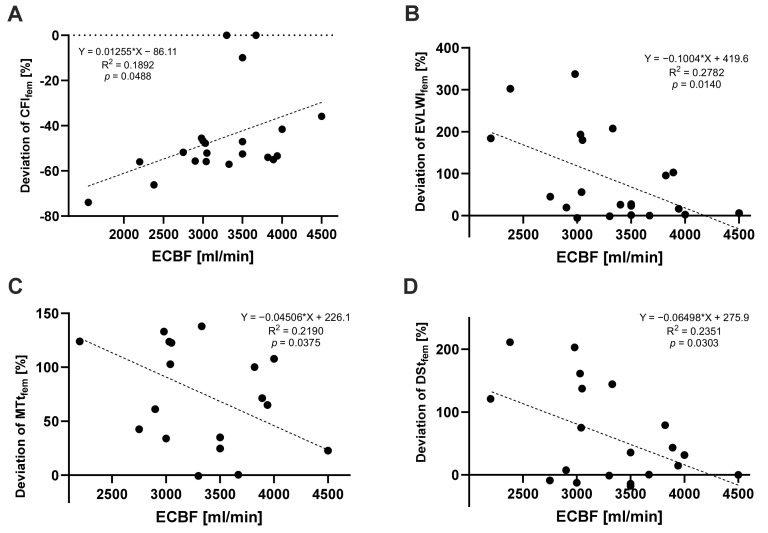
Simple linear regression model of the PiCCO-derived femoral deviations (as a percentage) from the PiCCO-derived jugular measurements as a function of the extracorporeal blood flow. (**A**) Deviation of CFI_fem_ (n = 21), (**B**) deviation of EVLWI_fem_ (n = 21), (**C**) deviation of MTt_fem_ (n = 20), and (**D**) deviation of DSt_fem_ (n = 20).

To illustrate the impact of ECBF on the differences between jugular and femoral measurements, we plotted the percentage deviation of femoral data in comparison to jugular data against ECBF (Figure 3). For CFI, EVLWI, MTt, and DSt, the deviation between femoral and jugular indicator injections became significantly smaller, with an increase in ECBF. Simple linear regression was performed and confirmed a significant correlation between the percentage deviation of the femoral from the jugular measurement and the ECBF for CFI (*p* = 0.0488, R^2^ = 0.1892), EVLWI (*p* = 0.0140, R^2^ = 0.2782), MTt (*p* = 0.0375, R^2^ = 0.2190), and DSt (*p* = 0.0303, R^2^ = 0.2351).

**Figure 4:**
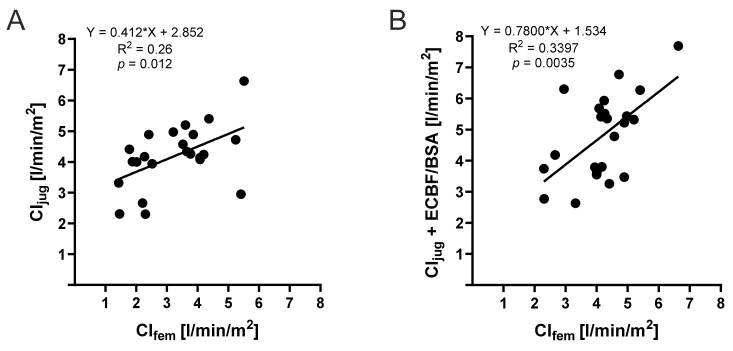
Simple linear regression between the femoral and jugular TPTD-derived cardiac index. (**A**) Simple linear regression (n = 23) between the femoral and jugular TPTD-derived cardiac index and (**B**) simple linear regression (n = 23) between the femoral and jugular TPTD-derived cardiac index plus extracorporeal blood flow indexed to body surface area.

As presented in Figure 1A, CI, after the jugular injection, significantly differed from CI after the femoral indicator injection, which can also be presented as deviation from the line of origin in a simple linear regression model (Figure 4A), indicating the weak comparability of both injection sites (R^2^ = 0.26). Interestingly, we observed a significantly improved correlation (*p* = 0.0035, R^2^ = 0.3397) when incorporating ECBF into the CI derived from the jugular indicator injection (Figure 4B). This not only highlights the substantial impact of ECBF on the disparity between femoral and jugular measurements but also offers a means to quantify this influence, thereby enhancing the interpretation of measured values.

## 4. Discussion

A recent prospective study focusing on the injection site for TPTD in patients undergoing vv-ECMO therapy [7] observed that the parameters measured in patients after femoral injections were significantly higher than those after jugular injections. Multivariate analyses revealed a significant independent correlation between increased GEDVI, EVLWI, and the femoral injection site [7]. Our results align with these findings, as we also observed significantly higher values for GEDVI and EVLWI in our femoral measurements compared to jugular measurements (Figure 1C,D). We found this difference across all cardiac function parameters, as well as volume parameters. The PiCCO^®^ device was chosen for thermodilution measurements because the patients were in respiratory failure. Other thermodilution methods, such as the pulmonary artery catheter, are of primary importance for patients with other forms of shock, such as cardiogenic shock [3]. The PiCCO^®^ data collected on EVLWI are of particular clinical, diagnostic, and therapeutic interest for these patients.

Regarding the CFI, we demonstrated that measurements via femoral injection in the same patient under ongoing vv-ECMO therapy resulted in a significantly lower values of CFI (femoral measurement, 1.44 ± 0.60 min^−1^ vs. jugular measurement, 2.53 ± 1.06 min^−1^). Previous studies in patients with ongoing ECMO therapy have already shown a significant difference between the CFI obtained from jugular vs. femoral measurements [5]. The differences in CFI can be explained by the mathematical relationship of the calculation (CFI = CO/GEDV). Other studies have shown that GEDV is significantly overestimated when the cold saline bolus of the thermodilution measurement is applied via femoral access [12,13]. This overestimation is due to a prolonged MTt with femoral injection, which is caused by the larger distance of the femoral catheter to the right atrium [13]. As a result, the CFI is systematically underestimated. Although a correction formula has been developed for the overestimated GEDV with femoral injections [12], the results of other parameters such as CFI cannot be corrected by formula use [5,14]. Therefore, in clinical practice, cardiac parameters such as CFI should be measured using other methods such as echocardiography in patients for whom only femoral access for TPTD measurement is available [5].

During ongoing extracorporeal therapy, a significant portion of the injected cold fluid bolus is lost as LOI through the extracorporeal circuit, which also influences TPTD values. In this context, in a pioneering study during the 1990s by Haller et al., a significant LOI was observed for the first time [15] with an overestimation of CO up to 300% [15]. These results were confirmed by studies with animal models [16]. Some of the differences in TPTD measurements under ECMO therapy can be explained by the underlying mathematical relations. An amount of fluid and the AUC are needed to calculate cardiac parameters, while MTt and DSt are used to calculate volume parameters such as EVLWI and GEDVI. A change in blood flow, as seen under ECMO therapy, affects the AUC, which serves as the basis for calculating other volume parameters using MTt and DSt [8]. Minini et al. proposed that the changes in TPTD measurements depend on the level of ECBF and the patient’s CO. Measurements deviate particularly under high ECBF and low patient CO [8]. By contrast, other studies concluded that TPTD measurements could remain plausible and credible when ECBF is no more than 20% of the patient’s CO [15,17]. The data from Minini et al. [8] align with our findings. Particularly, we observed that both MTt and DSt were significantly higher in femoral measurements compared to jugular measurements (Figure 1E,F). This can be explained by the increased distance between the thermistor-tipped catheter and the right atrium when femoral measurement is used, as well as the loss of a relevant proportion of the femoral-injected indicator into the ECMO circuit. It is worth noting that the literature on LOI in other extracorporeal support systems, such as renal replacement therapy, is not clear. Some studies found no relevant differences in PiCCO parameters under ongoing extracorporeal blood flow [17,18], while others suggest that even the lower extracorporeal volume of renal replacement therapy compared to ECMO blood flow may have an impact on the thermodilution method in patients with fever [19]. However, it should be noted that the studies by Sakka et al. were conducted in ICU patients with preserved cardiac output (CI: 3.8 ± 1.4 L/min/m^2^), and the extracorporeal blood flow was low (mean: 112.9 mL/min) [17]. Therefore, comparing CRRT with ECMO therapy, where significantly higher levels of ECBF are used, may not be appropriate. To demonstrate the influence of ECBF on the measurements, we correlated MTt and DSt with ECBF. We found a linear increase in MTt and DSt after an increase in ECBF. This aligns with the findings of Herner et al., who recorded significantly prolonged MTt and DSt in the thermodilution measurement after connection to vv-ECMO, compared to values recorded before the connection [7]. We further investigated the influence of ECBF on differences between jugular and femoral measurements. We correlated cardiac function parameters while considering the actual ECBF. Since femoral CO was significantly higher than jugular CO in the same patient, we created a mathematical model using CI_jug_ and the ECBF/BSA, which we compared with CI_fem_. We found a linear correlation (*p* = 0.0035) (Figure 4B). The level of ECBF appears to have a relevant influence on measurements via femoral injection site. To the best of our knowledge, this study is the first to relate ECBF to the patient’s BSA in relation to the recorded CI.

We hypothesized that ECBF must have a relevant impact on the transit times of the injected cold bolus. In accordance, Herner et al. were already able to show significantly prolonged MTt and DSt for the femoral injection of a cold bolus under vv-ECMO therapy compared to jugular measurements before and after ECMO connection [7]. With longer transit times through vv-ECMO, i.e., at lower ECBF, the time that the injected cold bolus is exposed to heating of the ECMO device is prolonged. This increases the temperature of the cold bolus and impairs its detection by the receiver. We postulated that slower ECBF is associated with greater differences between jugular and femoral parameters. The deviation of MTt and DSt after femoral vs. jugular injections significantly correlated with ECBF, resulting in significantly higher EVLWI and significantly lower CFI at lower ECBF via the femoral measurement compared to the jugular measurement.

## 5. Limitations

Our study has obvious and major limitations. The aim of our study was to directly follow the findings of Herner et al. [7], which are highly relevant for clinical practice, as they indicate a major impact of indicator injection site on TPTD measurements with the PiCCO^®^ device under vv-ECMO. We would like the data shown here to be interpreted as strong evidence that the effects of different injection sites can be detected in clinical practice. We are aware of the fact that a retrospective analysis that only includes measurements from two different patients has limited statistical power and should therefore only be considered hypothesis-generating.

For future studies, a significantly higher number of participants and more measurements considering the clinical circumstances are scheduled. TPTD measurements before connection to vv-ECMO as a baseline for interpretation as in Herner et al. [7] would also be beneficial. In addition to the values obtained by thermodilution, a correlation with echocardiographic examinations of the patient’s CO is missing. This should be an essential component for further analysis. A major challenge is the temperature measurement of the returning blood from vv-ECMO. Complex calorimetric measurements are necessary to investigate the influence of temperature change on the injected cold bolus.

Prospective, randomized studies are needed to investigate the influence of ECBF, actual CO determined by other diagnostic methods such as echocardiography, and the temperature change due to the device itself on the results of transpulmonary thermodilution depending on the injection site. Overall, a considered and critical approach to thermodilution under ongoing ECMO is highly recommended.

## 6. Conclusions

In this present retrospective study in a small sample of ICU patients with acute respiratory failure, we were able to indicate the influence of extracorporeal therapy on TPTD measurements and thus contribute to the understanding of the complexity of hemodynamic monitoring and its pitfalls. In a PiCCO^®^-derived hemodynamic assessment of patients on vv-ECMO, femoral indicator injections, as opposed to jugular injections, resulted in an overestimation of all index parameters. This discrepancy can be attributed to mean transit time (MTt) and downslope time-dependent (DSt) variations in GEDVI and cardiac function index and is correlated with ECBF. Due to extracorporeal circulation and temperature changes caused by vv-ECMO therapy, differences in TPTD-derived readings occur depending on the site of injection. Femoral injections lead to an overestimation of the parameters compared to jugular injections, which could be due to longer MTt and DSt. A linear correlation can be shown between the CI of the different injection sites, considering the ECBF indexed to the BSA.

## Figures and Tables

**Table 1:** Demographic and clinical characteristics. *ABPd*, diastolic arterial blood pressure; *ABPs*, systolic arterial blood pressure; *BMI*, body mass index; *BSA*, body surface area; *mCVP*, mean central venous pressure; *ECBF*, extracorporeal blood flow; *HR*, heart rate.

Characteristics	(Mean ± SD, Percentages)
Sex [male:female, n]	0:2
Age [years]	57.50 ± 3.54
Weight [kg]	87.50 ± 31.82
BMI [kg/m^2^]	28.22 ± 8.10
BSA [m^2^]	2.02 ± 0.38
**Measurements (n = 28)**	
ABPs [mmHg]	101.00 ± 36.77
ABPd [mmHg]	47.47 ± 18.03
HR [bpm]	67.07 ± 26.47
sinus rhythm [%]	100
mCVP [cmH_2_O]	8.37 ± 4.36
ECBF [L/min]	3.24 ± 0.65
Mechanical ventilation [%]	100
Norepinephrine [%]	74.19
Norepinephrine [µg/kg/h]	5.26 ± 19.43
**Comorbidities**	
Renal replacement therapy [n]	0/2
Valvular diseases [n]	0/2
Coronary heart disease [n]	0/2
Peripheral artery disease [n]	0/2

## Data Availability

All data are presented in the manuscript.

## Supplementary Materials

The following supporting information can be downloaded at https://www.mdpi.com/article/10.3390/jcm13082334/s1, Table S1: Comparison of TPTD-derived parameters from jugular and femoral indicator injections.
