# Supplementary material for: Injection Site Matters: A Comparative Analysis of Transpulmonary Thermodilution via Simultaneous Femoral and Jugular Indicator Injections under Veno-Venous Extracorporeal Membrane Oxygenation Therapy
Source: J Clin Med. 2024 Apr 17;13(8):2334. doi: 10.3390/jcm13082334 (PMC11050890; doi:10.3390/jcm13082334)
Supplement: Supplementary file 1 [file jcm-13-02334-s001.zip › jcm-2903943-supplementary.pdf]

Comparison of TPTD-derived parameters

**Parameter**	**Jugular indicator injection**	**Femoral indicator injection**	**Number of pairs**	**P-value**
**CO [l/min]**	6.37 ± 2.11	8.31 ± 2.07	23	**0.0029**
**CI [l/min/m^2]**	3.10 ± 1.19	4.19 ± 1.01	23	**0.001**
**CFI [1/min]**	2.53 ± 1.06	1.44 ± 0.60	21	**< 0.0001**
**SVRI [dynSm^2/cm^5]**	1861 ± 771	1303 ± 471	23	**0.005**
**EVLW [ml]**	1250 ± 499	1931 ± 635	21	**0.0002**
**EVLWI [ml/kg]**	18.3 ± 6.71	29.3 ± 10.9	21	**0.0003**
**ITBV [ml]**	1587 ± 332	3923 ± 1193	21	**< 0.0001**
**ITBVI [ml/m^2]**	806 ± 125	2163 ± 631	21	**< 0.0001**
**GEDV [ml]**	1260 ± 267	3139 ± 955	21	**< 0.0001**
**GEDVI [ml/m^2]**	687 ± 141	1731 ± 505	21	**< 0.0001**
**PVPI**	3.89 ± 1.47	2.49 ± 1.21	21	**0.004**
**SVV [%]**	14.1 ± 5.56	12.7 ± 5.45	22	0.07
**ITTV [ml]**	2837 ± 729	5897 ± 1387	20	**< 0.0001**
**PTV [ml]**	1616 ± 616	2597 ± 883	20	**0.0002**
**MTt [s]**	29.50 ± 11.80	45.40 ± 16.60	20	**< 0.0001**
**DSt [s]**	16.70 ± 8.21	21.0 ± 7.83	20	**0.0031**

Supplementary Table S1: Comparison of TPTD-derived parameters from jugular and femoral indicator injections

CI cardiac index, *CFI* cardiac function index, *CO* cardiac output, *DSt* downslope time, *EVLW* extravascular lung water, *EVLWI* extravascular lung water index, *GEDV* global end-diastolic volume, *GEDVI* global end-diastolic volume index, *ITBV* intrathoracic blood volume, *ITBVI* intrathoracic blood volume index, *ITTV* intrathoracic thermal volume, *MTt* mean transit time, *PTV* pulmonary thermal volume, *PVPI* pulmonary vascular permeability index, *SVRI* systemic vascular resistance index.
